# Local Health Departments Addressing the Social Determinants of Health: A National Survey on the Foreclosure Crisis

**DOI:** 10.1089/heq.2018.0066

**Published:** 2019-02-21

**Authors:** Katherine Schaff, Lori Dorfman

**Affiliations:** ^1^ Alameda County Public Health Department, Oakland, California.; ^2^ Berkeley Media Studies Group—A Project of Public Health Institute, Berkeley, California.

**Keywords:** foreclosure, health equity, housing, local health departments

## Abstract

**Purpose:** To examine local health department (LHD) engagement in addressing the social determinants of health by using the foreclosure crisis as an example.

**Methods:** National survey of 166 LHD staff on the foreclosure crisis (2006–2014).

**Results:** About one quarter (28%) of respondents reported that their LHD had engaged in work related to the foreclosure crisis, 7% planned to engage, and 65% did not or were not planning to engage. Views about the role of LHDs in addressing the foreclosure crisis varied: 30% stated that LHDs should work on foreclosure.

**Conclusions:** A substantial number of respondents reported that their LHD addressed foreclosure, or supported engagement, yet there are divergent perceptions of appropriate LHD roles. LHDs follow a pattern described by the diffusion of innovations theory: Innovative LHDs can share their work on foreclosure and housing, early adopters are poised to act, and others may follow if they have support.

## Introduction

The foreclosure crisis drastically reshaped access to housing and wealth in the United States, with widespread health impacts for homeowners, renters with landlords facing foreclosure, and communities with high rates of foreclosure.^1,2^ A homeowner going through foreclosure or a renter whose landlord undergoes foreclosure faces housing instability, a disruption of social networks, and increased financial burden—all of which are associated with negative physical and mental health outcomes, such as hypertension, heart disease, and depression.^3–10^ Communities with high rates of foreclosure often face elevated rates of violent crime and associated poorer health outcomes.^5–8^ Related to patterns of segregation, disinvestment, predatory lending, and decades of policies that harm communities of color and lower-income communities, the foreclosure crisis disproportionately affected lower-income, African American, Latinx, and some Asian and Pacific Islander communities and stands to exacerbate racial and class inequities in health for current and future generations.^11–13^

Local health departments (LHDs) are increasingly involved in addressing social determinants of health, such as housing, and supporting communities in building power as strategies to achieve health equity.^14,15^ LHDs can play a unique role in addressing the social determinants of health through: leadership and convening, access to data and decision makers, partnerships with residents and community organizations, mandates and legal authority, and by connecting social factors and health.^16–19^ Housing is a core social determinant of health, and we would accordingly expect LHDs to be responsive to emerging and pressing foreclosure-related concerns and health impacts within their jurisdictions. However, there has been little research on whether and how LHDs engage in addressing housing and foreclosure as determinants of health. In this research, we begin to address this gap by reporting on a national survey that explores LHD engagement, roles, and responsiveness to the foreclosure crisis.

## Methods

To understand the response of LHDs in the United States to the foreclosure crisis (2006–2014), we designed, pilot-tested with staff from a diverse range of LHDs, and administered an online Qualtrics survey with closed and open-ended questions. Using skip logic, the survey ranged from 5–10 questions regarding ongoing or potential foreclosure-related work, barriers to engagement, and perceptions about whether LHDs should be addressing the foreclosure crisis. The Institutional Review Board at the University of California, Berkeley, approved this study protocol. Survey respondents provided informed consent.

We invited all LHDs in the United States (∼2800) by using the National Association of County and City Health Official's LHD Index, and numerous listservs, regional and state public health associations, and personal contacts. To increase the response and completion rate, respondents qualified for a drawing for one of two $25 gift cards. More than one respondent from each LHD could participate because: we wanted to capture diverse perspectives; staff may know about different work happening within their LHD depending on their job and how large the health department is; and we were focused on exploratory research to gather perceptions rather than statistically generalizing responses to all LHDs. We considered surveys to be complete when respondents advanced through each page and clicked on the link to take them to a final question about participating in the raffle. However, the N varies per question based on (1) skip logic; (2) some questions were optional; and (3) some respondents chose not to answer specific questions.

One author and another researcher coded all responses by using Qualtrics to analyze close-ended questions and Dedoose for inductive/deductive coding of open-ended questions to identify major themes, resolving differences through consensus.^20,21^

## Results

There were 166 completed surveys from 159 LHDs in 36 states; 7 LHDs had 2 respondents each (Table 1). Respondents were from a mix of township, city, county, and regional LHDs, with the smallest serving 712 residents and the largest serving 9,818,696 residents, based on 2010 population estimates of the U.S. Census Bureau (Table 2). Twenty-eight percent of respondents said that their LHD had engaged in work related to the foreclosure crisis, 7% noted that their LHD was planning to engage in the next year, and 65% reported that their LHD was not engaging or planning to engage in work related to foreclosure (Table 3, *n*=166). Thirty percent believed that their LHD should work on foreclosure, 46% thought that their LHD should not engage, and 24% selected “other” with the option to provide additional comments, many of which described barriers and challenges to the work (*n*=160).

**Table 1.** Local Health Department Survey Respondents by State

No. of respondents	State
0	Alabama
	Alaska
	Delaware
	Georgia
	Hawaii
	Nevada
	New Hampshire
	New Mexico
	Rhode Island
	South Carolina
1	Idaho
	Indiana
	Louisiana
	Maine
	Maryland
	Michigan
	Mississippi
	Missouri
	Nebraska
	Oklahoma
	Pennsylvania
	South Dakota
	Utah
	Vermont
	Wyoming
2	Arkansas
	Connecticut
	Oregon
	Tennessee
	Virginia
3	Arizona
	Florida
	Kentucky
	North Carolina
	North Dakota
	West Virginia
4	Montana
5	Iowa
	Kansas
	New Jersey
	New York
6	Colorado
	Washington
7	Illinois
	Minnesota
11	California
12	Wisconsin
14	Texas
17	Massachusetts
19	Ohio

**Table 2.** Size of Population Served by the Local Health Departments of Survey Respondents

Size of population served	No. of respondents	%
<10,000	21	12.65
10,000–24,999	29	17.47
25,000–49,999	23	13.86
50,000–74,999	16	9.64
75,000–99,999	17	10.24
100,000–199,999	21	12.65
200,000–499,999	16	9.64
500,000–999,999	13	7.83
1,000,000+	10	6.02
Total	166	

Due to rounding, percentages may not add up to 100%.

Population data used for analyses are based on 2010 population estimates of the U.S. Census Bureau.

**Table 3.** Survey Responses from Local Health Department Staff to “Has Your Health Department Engaged in Any Work in Response to the Foreclosure Crisis (2006–2014)?” Classified by Mutually Exclusive Diffusion of Innovation Categories (*n*=166 Respondents)

Potential diffusion of innovations category	Answer	Response	%
Innovators/early adopters	Yes	47	28
Early/late majorities	No, we have not engaged in any work related to foreclosure, but are planning on engaging in work related to foreclosure in the next year. Please describe what type of work you will be engaging in:	11	7
Laggards^a^	No, we have not engaged in any work related to the foreclosure crisis and do not plan on engaging in work related to foreclosure.	108	65
	Total	166	100

^a^ This language comes from Diffusion of Innovations theory. Health departments in this category may have compelling reasons for not engaging in the foreclosure crisis, such as budget and staffing cuts or other emerging threats.

Of the respondents who answered questions about how they engaged (*n*=43), meeting with other city and county agencies to discuss and plan responses to the foreclosure crisis (44%) and communicating with community organizations that address foreclosure (40%) were the most common responses (Table 4). Just more than one-third advocated for local polices related to foreclosure (35%), and one-third contacted local banks and finance groups (33%). The majority (64%, *n*=41) said there were fewer than five staff working on the issue.

**Table 4.** Survey Responses from Local Health Department Staff to “You Indicated Your Health Department Has Engaged in Work in Response to the Foreclosure Crisis (2006–2014)”^a^

Answer	Response	%
Met with other city or county agencies to discuss and plan responses to the foreclosure crisis	19	44
Other: Please describe	18	42
Communicated with community organizations that are addressing foreclosure	17	40
Advocated for local policies related to foreclosure.	15	35
Reached out to/communicated with local banks/finance groups regarding the foreclosure crisis	14	33
Communicated with the media about the impact of foreclosure on health	10	23
Provided data or research regarding the health impacts of foreclosure to partners, policymakers, advocates, or the media	7	16
Released reports, factsheets, articles, blogs, or other information on foreclosure and health, either independently or in collaboration with other organizations	7	16
Coordinated physical and/or mental health service provision with organizations providing foreclosure counseling or other services	7	16
Provided targeted physical and/or mental health services to those facing foreclosure	5	12
Provided formal testimony or communicated to policymakers regarding the health impact of the foreclosure crisis	5	12
Analyzed local lending patterns in communities affected by the foreclosure crisis	2	5

*n*=47/166 respondents were eligible to answer this question because of skip logic; responses not mutually exclusive.

^a^ This question only appeared in surveys for respondents who said their health department engaged in work related to the foreclosure crisis. Longer descriptions/examples of each activity were given in the survey.

Respondents described seven roles for LHDs, from no engagement to deeply engaged (Table 5; *n*=165 responses). Eight responses (4.8%) suggest no role for LHDs because other social determinants, such as poverty, were a more urgent local priority or because foreclosure had not been raised by clients during clinic visits. Fourteen responses (8.5%) indicate that LHDs could have a role but that it is unclear what it should be. More responses (35 or 21.2%) define the role of LHD as connecting clients to services and case management to mitigate the impacts of foreclosure on individuals. Most responses (47 or 28.5%) connect the role of LHD to environmental health impacts, specifically mandated and regulatory activities such as addressing nuisance complaints to holding banks accountable for property maintenance. Nine (5.5%) responses describe a role for LHDs in conducting community assessments or data analysis related to foreclosure. Twenty-two (13.3%) responses define partnering with others as a key role. Finally, 30 responses (18.2%) suggest that LHDs should address foreclosure as a social determinant of health that drives health inequities.

**Table 5.** Survey Respondents' Perceptions About the Role of Local Health Departments in Addressing the Foreclosure Crisis (2006–2014) Moving from No Role to Deeply Engaged (*n*=165 Coded Responses)

Perception of LHD role	No. of responses that describe this role (not mutually exclusive)	Examples and quotes
No role: Did not think it was necessary for LHDs to address foreclosure.	8	• “It's not a part of our mission nor is it considered core public health. Our hope is this issue would be addressed by other, more appropriate agencies.”
		• “We are located in Appalachia where poverty has always been an issue. The foreclosure crisis hasn't had a significant impact on chronic poverty issues.”
Unclear role: Believe LHDs could have a role, but unclear what that role should be.	14	• “We are just beginning to explore ways to more directly address upstream, root causes for some of the health challenges we see. Public health in general is upstream, but we are trying to determine just how far upstream we should go.”
Connectors: LHDs should connect clients to services, health care, and case management.	35	• “Other than referring clients to programs that are designed to assist, we have so much other work. We can only advocate for more assistance.”
Address environmental issues: LHDs should focus on environmental health and safety related to housing.	47	• “Our sewage code requires us to take legal action when property owners fail to follow the code.”
		• “We have found putting the pressure on the bank that holds the mortgage does help with enforcement to secure the property and maintain the property so that it is safe.”
Conduct assessments: LHDs should conduct community assessments or data analysis.	9	• “Working on doing a community health assessment and community health improvement plan; foreclosure would be part of that.”
Partner with other organizations: LHDs should work with other government and/or community-based entities.	22	• “It depends on other factors. If we were putting together a city-wide team of key departments, health should be part and I would welcome this opportunity.”
Address social determinants of health, including foreclosure: LHDs should address social factors that affect health, such as foreclosure and housing.	30	• “In a sense, it is a community disaster. Responding to such disasters and crises to preserve and restore health is a core public health function.”
		• Many respondents included terms such as “economics,” “low-income communities,” “poverty,” or “certain communities.” Only one respondent used the term “communities of color,” which was the only comment in this category that more explicitly raised racial inequities related to foreclosure.

Responses are not mutually exclusive. For example, one response may be coded as partnering with other organizations as well as conducting assessments.

LHD, local health department.

## Discussion

The foreclosure crisis, and more broadly, housing, will continue to affect public health broadly, including racial and economic disparities in health.^22,23^ As little research has examined LHD staff perceptions about the role of LHDs in addressing social determinants of health, such as foreclosure or housing, the wide variation in both opinions on appropriate roles for LHDs and current LHD work related to foreclosure is a significant finding.

Diffusion of innovations theory describes how new ideas or practices are adopted within or across organizations.^24^ This research suggests that LHDs are moving through a diffusion of innovations process, with fewer innovators and early adopters engaging in addressing foreclosure and housing as one strategy in tackling health inequities; early and late majority LHDs contemplating engagement; and other LHDs with no plans for engagement (Table 3). Although fewer LHDs are working on policy change related to foreclosure, these results demonstrate that innovative LHDs, despite challenges, are expanding the boundaries of local public health practice and developing new roles and strategies for LHDs to address social determinants of health, such as the foreclosure crisis. Implications for practice include developing case studies or resources based on lessons from innovative LHDs and better understanding and addressing barriers that LHDs face.

The results led us to question why some innovative LHDs have addressed foreclosure, specifically through policy and community partnerships, whereas others have yet to engage. Future analysis of these data will examine the variability in responses across LHDs, including factors that facilitate innovation as well as barriers that LHDs face in addressing the foreclosure crisis, and will likely be relevant to LHDs working to address other social determinants of health.^25^

### Limitations

Although this study illuminates the role of LHD in addressing the foreclosure crisis, it has several limitations. It is not representative of LHDs in the United States nor generalizable. The LHD staff who self-selected to take the survey may differ in significant ways from staff who did not take it, and the work that their LHD does may also differ. Survey responses were brief and do not reflect the full knowledge or perceptions of respondents. Finally, there are limitations to survey methodology, such as the influence of wording and the order of questions, and specific limitations related to online surveys. However, given the lack of research on LHD engagement in addressing social determinants of health, this survey helps build the foundation for future research and action.

## Conclusion

As a growing number of LHDs tackle the root causes of health inequities through addressing the social determinants of health such as housing and foreclosure, research on challenges, opportunities, and LHD staff views of their own role in confronting these issues is essential to achieving health equity.

Catalyzing and accelerating innovation across LHDs is a critical step in advancing public health practice focused on addressing the social determinants of health. Our results indicate there are innovative LHDs that can share lessons learned and best practices, early adopters ready to take on this work, and early and late majority LHDs who may be able to soon follow if challenges are met and they are provided with support. Future research on the characteristics and approaches that LHDs have and use, the systems and contexts they work within, and the barriers they face can provide clear, concrete steps for advancing public health practice and ensuring that LHDs use their unique role to help the country move toward racial and health equity.

## Abbreviation Used

LHD: local health department
